# 

**Published:** 1894-03-24

**Authors:** 


CONTENTS.

London Ambulance: "Benefits Forgot" - 451
Around the Hospitals - 452
On Modern Progress of Ophthalmic Medicine and Surgery. By R. BRUDENELL CARTER, F.R.C.S. - 453
The Educational Treatment of Idiots, Imbeciles, and Feeble-Minded Children. By G. E. SHUTTLEWORTH, B.A., M.D., &c. - 454
Studies in Applied Sociology. V.—The Poor and the Poor Law - 455
The Field of Science - 456
Annotations. — Chelsea and the Preventive Institute — Mr. Gladstone's Eyesight—Dr. Louis Parkes: A New Surgical Censorship - 457
Notes and News - 458
Medical Progress and Hospital Clinics—
The Treatment of Pneumonia - 459
A Case of Neuroma of the External Popliteal Nerve - 460
Edinburgh Royal Infirmary—Treatment of Acute Rheumatism 461
MEDICAL PROGRESS.—Malaria - 462
Rhinology - 463
NEW DRUGS AND PREPARATIONS - 465
NEW APPLIANCES AND THINGS MEDICAL - 466
The Institutional Workshop—
HOSPITAL FINANCE. IV.—Voluntary Offerings (*continued*) - 467
PRACTICAL DEPARTMENTS - 468
HOSPITAL FESTIVALS, MEETINGS, &C. - 469

EXTRA SUPPLEMENT.—THE NURSING MIRROR.

News from the Nursing World.—The Royal British Nurses' Association—The Royal National Pension Fund for Nurses—District Nursing at Richmond—A Warning to Nurses—"On Duty Night and Day"—Girl Nurses Again—Bristol Nurses Training Institute—An Urgent Appeal—The Senior Nurse—Queen's Nurses at Aberdeen—Nursing at Ayr—Nuns at Chicago—Another New Nurses' Home—A Valued Army Nurse—A Faithful Nurse—Short Items - ccxli

On General Nursing. IV.—Scarlet Fever (*continued*) - ccxliii

Lectures at St. George's Hospital - ccxliv

The Royal National Pension Fund for Nurses - ccxlv

The Difficulties of Private Nursing. By ONE WHO KNOWS THEM - ccxliv

Guy's Hospital Trained Nurses' Institute - ccxlvii

What a Nurse Should Read - ccxlviii

Novelties for Nurses - ccxlviii

"Nervous Heredity in Villages" - ccxlix

Queen's Nurses at Worthing—Where to Go - ccxlix

Presentation - ccxlix

Everybody's Opinion - ccl

Wants and Workers - ccl

For Reading to the Sick - ccl

